# Pulmonary metastases from primary hepatocellular carcinoma in a 26-year-old patient: a case report

**DOI:** 10.4076/1757-1626-2-6256

**Published:** 2009-08-10

**Authors:** Carla Assed, Edson Marchiori, Gláucia Zanetti, Claudia Mauro Mano, Branca Sarcinelli-Luz, Flávia Gavinho Vianna, Juliana França Carvalho, Isabella Guedes Santos, Alair Augusto Santos, Alberto Domingues Vianna

**Affiliations:** Department of Radiology, Faculty of Medicine, Fluminense Federal UniversityRio de Janeiro, Rua Thomaz Cameron, 438, Valparaiso CEP 25685.120Brazil

## Abstract

Hepatocellular carcinoma is a primary tumor of the liver, which usually develops in the setting of chronic liver disease, particularly viral hepatitis. The diagnosis of hepatocellular carcinoma can be difficult, and often requires the use of serum markers, one or more imaging modalities, and histological confirmation. The authors describe a case of a 26-year-old woman with hepatocellular carcinoma and multiple pulmonary metastases. She presented with hepatomegaly and sporadic fever, and had negative hepatitis serology, normal alkaline phosphatase, and a rising serum alpha-fetoprotein level. The diagnosis was confirmed by histopathology, after percutaneous liver biopsy. Although the patient was in good health condition and had few symptoms, there was no possibility of treatment due to the extension of the liver tumor and the number of pulmonary metastases.

## Introduction

Hepatocellular carcinoma (HCC) is the main primary hepatic tumor and one of the most common cancers worldwide [1]. It is usually seen at the sixth and seventh decades of life. Men are affected three times as often as women, and blacks are affected twice as often as whites [2]. The main risk factors associated with HCC are hepatitis C virus (HCV) or hepatitis B virus (HBV) infection and alcoholic cirrhosis; chronic hepatitis B is the most frequent cause [3]. Some cases are also associated with exposure to environmental carcinogens such as aflatoxin [2]. Intra-hepatic recurrence is more frequent but pulmonary metastasis is the chief site of extrahepatic spread, followed by regional and distant lymph nodes, musculoskeletal system, adrenal glands [4], kidneys and bone marrow [3]. HCC has a dismal overall prognosis, with more than 90% of affected individuals dying of the disease [4]. Several methods of treatment for HCC are often used in combination for either palliation or cure. Multiple modalities are available to treat HCC, including chemotherapy, liver resection, liver transplantation, and ablative therapies such as radiofrequency ablation, transarterial embolization and percutaneous ethanol injection [5,6]. The suitable treatment depends on the stage of the tumor at the moment of diagnosis. When pulmonary metastases are present, surgical resection can still be an option in some cases. We report a case of HCC with pulmonary metastases and no possibility of surgical treatment.

## Case presentation

A 26-year-old female Brazilian Caucasian patient was admitted to the hospital presenting with a one-month history of right-sided pleuritic chest pain, nausea, two episodes of hemoptysis and fever. There was no history of weight loss during this period. Physical examination was unremarkable, except for a hepatosplenomegaly and diminished vesicular breath sounds over the right lung base.

Laboratory evaluation revealed a red blood cell count of 3.89 × 10^6^ cells/mm^3^; hemoglobin, 10.5 g/dL; hematocrit, 31.5%; MCV, 81%; MCHC, 33.5%; platelet count, 298,000/mm^3^, and normal white blood cell and differential count. Erythrocyte sedimentation rate was 107 mm, and C-reactive protein levels were 13.97 mg/dL. Serum AST and gamma-GT levels were 70 U/mL and 173 U/L, respectively; ALT, alkaline phosphatase and bilirubin levels were within the normal range. Hepatitis B and C virus serology tests were negative, CEA was normal, and alpha-fetoprotein level was above 300 UI/ml.

**Figure 1.**
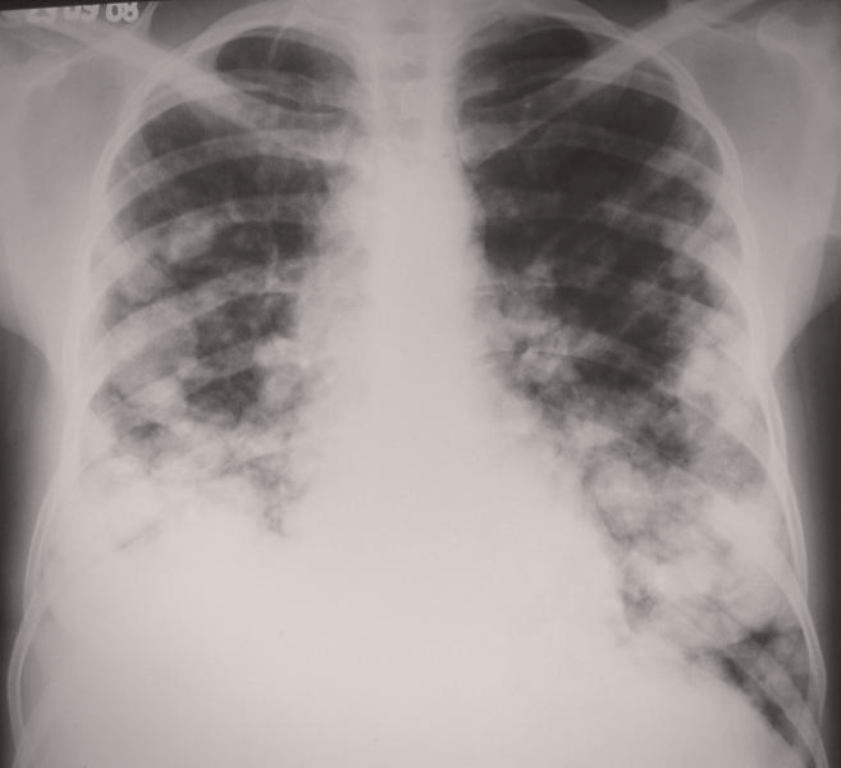
Chest X-ray showing multiple variable sized nodules predominating in the inferior areas of the lungs.

**Figure 2.**
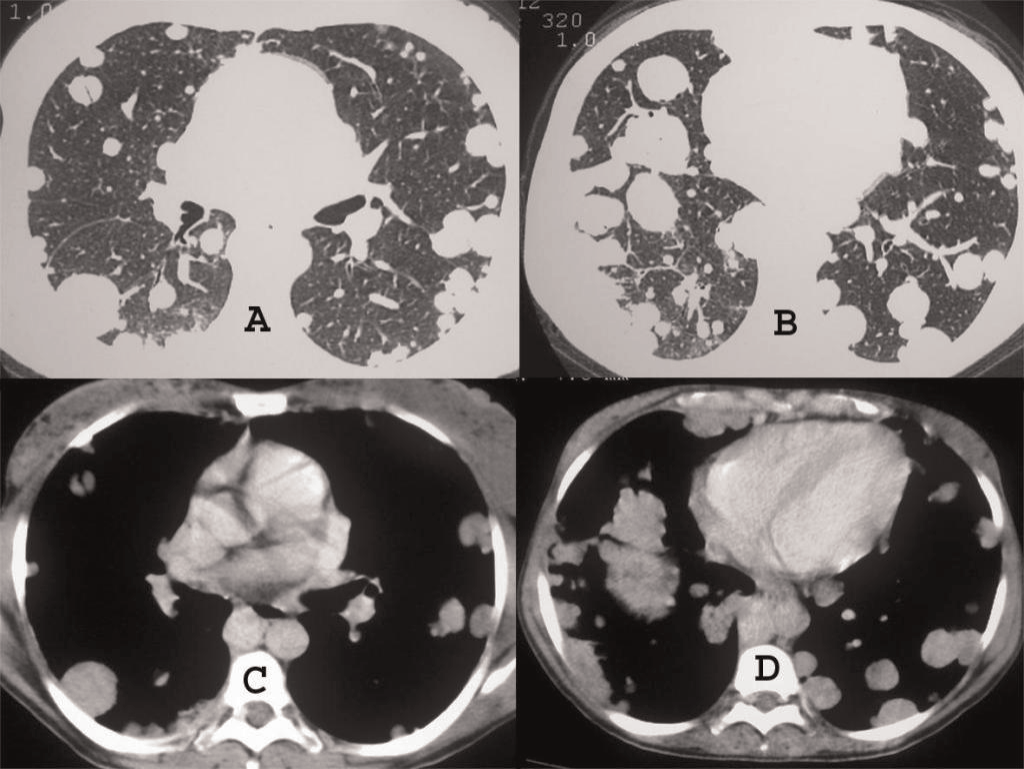
Computed tomography of the lungs, with pulmonary **(A and B)** and mediastinal **(C and D)** window settings, showing well-defined nodules, in a peripheral distribution.

**Figure 3.**
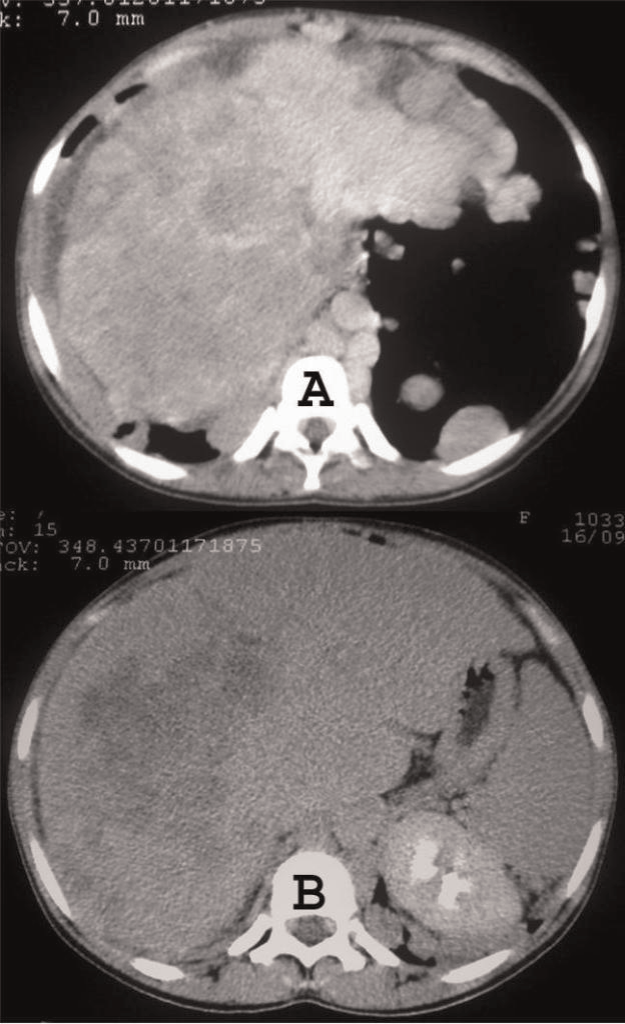
Computed tomography scans of the upper abdomen showing hepatomegaly, with an extensive ill-defined heterogeneous lesion in the hepatic parenchyma.

Chest radiograph demonstrated multiple variable sized nodules in both lungs (Figure 1). Abdominal ultrasound showed an enlarged liver with multiple hyperechoic nodules. Chest computed tomography (CT) revealed multiple well-defined nodules in a peripheral distribution (Figure 2), and computed tomography of the abdomen showed an enlarged liver with lobulated contours and an extensive heterogeneous lesion (Figure 3).

Subsequently, liver and pleural biopsies were performed, and confirmed the presence of a primary hepatocellular carcinoma and multiple pulmonary metastases. There were no signs of associated cirrhosis. Since the disease was at a very advanced stage, she was offered palliative chemotherapy. The patient had a poor response to treatment and died three months later.

## Discussion

Hepatocellular carcinoma is a common malignancy in patients with chronic hepatitis. In fact, HBV and HCV infection are the main etiologic factors for this neoplasm. The tumor frequently metastasizes via the lymphatic system, intrahepatic blood vessels or direct infiltration [3]. The lung is the most common site for extrahepatic spread, and its involvement is associated with a poor prognosis [1]: during necropsy, 52% of patients with primary liver carcinoma had evidences of pulmonary metastases [7].

Since most pulmonary metastases are multiple, they are often unresectable [1]. However, pulmonary metastasectomy can be indicated in cases where lesions are smaller than 3 cm and limited to the lungs [4]. In order to be eligible, the patient must be a good risk for surgical intervention [1], and some factors including the number and location of the lesions must also be considered [4].

Several imaging modalities are available for detection and characterization of HCC and metastatic lesions. These include ultrasonography, CT, magnetic resonance imaging and positrons emission tomography. Contrast-enhanced CT has high sensitivity (93%) and specificity (100%) for detecting hepatic metastases [8].

Since our patient presented an extensive liver lesion and multiple pulmonary nodules, surgical treatment was discarded and she was started on palliative chemotherapy. This case report describes an unusual presentation of HCC in a young patient without chronic hepatitis. Although she had few symptoms at the moment of diagnosis, her tumor was already at an advanced stage, with multiple pulmonary metastases.

## Abbreviations

CT: computed tomography
HBV: hepatitis B virus
HCC: hepatocellular carcinoma
HCV: hepatitis C virus
